# Why should Cannabis be Considered Doping in Sports?

**DOI:** 10.3389/fpsyt.2013.00032

**Published:** 2013-05-15

**Authors:** Mateus M. Bergamaschi, José Alexandre S. Crippa

**Affiliations:** ^1^Department of Neuroscience and Behavior, Ribeirão Preto Medical School, University of São PauloRibeirão Preto, São Paulo, Brazil; ^2^National Institute for Translational Medicine, CNPqPorto Alegre, Brazil

Recent debate and cases involving elite athletes raised the question whether or not *Cannabis sativa* (cannabis) should be considered doping in sports. Results from a 2010 report in the United States (Substance Abuse and Mental Health Services Administration, 2011) showed that cannabis is the most used illicit drug, with 17.4 million users smoking cannabis and 6.9 million users smoking cannabis on a daily or near daily basis. The World Anti-Doping Agency (WADA) included cannabis in its Prohibited List in 2004, claiming that cannabis may improve performance in some sports and is an illegal drug in most countries (Huestis et al., 2011); however, the inclusion of a substance in the Code (World Anti-Doping Agency, 2009) is complex, requiring intense debate among delegates and the fulfillment of specific criteria. For instance, Section 4 of the Code establishes that a substance be considered for inclusion in the Prohibited List if it is a masking agent or meets two of the three following criteria: (i) potential to enhance performance in sports – smoked cannabis affects cognition and performance, causes memory loss, executive function, and motor impairment, among other undesirable effect (Saugy et al., 2006). Cannabis smoking can be helpful for some activities such as extreme sports, as it improves muscle relaxation, reduces anxiety, and extincts fear memories (e.g., negative experiences) leading to enhanced performance. It is also worthwhile to note that cannabis smoking improves sleep time and recovery, which may favor performance when an athlete is facing multiple competitions in a short period of time. In light of these positive effects, one can assume cannabis is a doping substance that relaxes the mind and improves recovery (Huestis et al., 2011); (ii) potential or actual health risk – cannabis’ cognitive effects in chronic users are still unclear, but it may downregulate CB1 receptors, affect executive functions, and cause motor impairment, reversed only after weeks of abstinence (Hirvonen et al., 2012). It seems unlikely that athletes are chronic cannabis smokers due to the detrimental effects of chronic use including inconsistent performance, concentration, and motivation. Cyclists who smoked cannabis had a 1-min decrease in maximal exercise performance at 10 min after smoking (Renaud and Cormier, 1986). These negative effects on cognition and performance can impair critical skills (e.g., decision making, vigilance, alertness) required in high-risk sports to avoid accidents and/or injuries; or (iii) violation of the spirit of sport – doping is essentially contrary to the spirit of sport, which is the principle of Olympism, characterized by several values, such as ethics, fair play and honesty, health, respect for rules and laws, and respect for self and other participants (World Anti-Doping Agency, 2009).

Over 60 cannabinoids are present in cannabis, with Δ9-tetrahydrocannabinol (THC) the main psychoactive constituent and responsible for the observed toxic effects after smoking, while other cannabinoids are responsible for minor effects, such as cannabinol (CBN), which is 10% as psychoactive as THC (Huestis, 2005). THC is lipophilic and stores in several organs, especially in adipose tissue; this extensive body burden explains the prolonged cannabinoid detection rate in blood and urine for at least 4 weeks in chronic daily cannabis smokers (Lowe et al., 2009; Bergamaschi et al., 2013). The WADA (World Anti-Doping Agency, 2013) establishes a 15 ng/mL urinary 11-nor-9-carboxy-THC (THCCOOH) threshold; urine analyses involves THCCOOH-glucuronide conjugates cleavage, which significantly increases free THCCOOH concentrations and detection time. Urinary THCCOOH concentrations above the 15 ng/mL threshold are considered Adverse Analytical Findings and may be interpreted as a violation of anti-doping rules (World Anti-Doping Agency, 2009). Studies showed that even occasional and single cannabis smoking might yield a THCCOOH positive result (≥15 ng/mL) for up to 5 days (Huestis et al., 1996). Thus, consuming cannabis even weeks before a match may imply a considerable risk of being detected in a doping test. In light of this considerable risk, some users started using a new preparation of herbal smoking blends named “Spice.” Such substances are highly potent cannabinoid analogs, with unknown and potentially harmful toxicological properties that may cause prolonged intoxication. These substances mimic or worsen cannabis’ toxic effects provoking cognitive and motor impairment (UNODC, 2011).

The non-psychoactive cannabidiol (CBD) is anxiolytic in humans following a single dose (Zuardi et al., 1993; Bergamaschi et al., 2011); decreased anxiety and fear memories extinction after oral CBD intake may enhance sports performance with no “violation” of the Code, as no THCCOOH is detected in urine. One way to protect athletes’ health and to promote health, fairness, and equality in sports is to include any illicit drugs, their constituents and analogs in the anti-doping program. The sports may assist to create educational program for youth and athletes as an alternative to keep them away from drugs and to preserve the intrinsic value about the “spirit of sport.”
